# Validation of the Telephone-Administered Version of the Mediterranean Diet Adherence Screener (MEDAS) Questionnaire

**DOI:** 10.3390/nu12051511

**Published:** 2020-05-22

**Authors:** Maria João Gregório, Ana M. Rodrigues, Clara Salvador, Sara S. Dias, Rute D. de Sousa, Jorge M. Mendes, Pedro S. Coelho, Jaime C. Branco, Carla Lopes, Miguel A. Martínez-González, Pedro Graça, Helena Canhão

**Affiliations:** 1 Comprehensive Health Research Centre (CHRC), NOVA Medical School, UNL, 1099-085 Lisboa, Portugal; mariajoaobg@gmail.com (M.J.G.); ana.m.rodrigues@nms.unl.pt (A.M.R.); salvadorclf@gmail.com (C.S.); rute.sousa@nms.unl.pt (R.D.d.S.); jaime.branco@nms.unl.pt (J.C.B.); 2 EpiDoC Unit, Centro de Estudos de Doenças Crónicas (CEDOC) da NOVA Medical School, Universidade Nova de Lisboa (NMS/UNL), CEDOC—Campus Sant’Ana, Pólo de Investigação, NMS, UNL, Edifício Amarelo, Rua do Instituto Bacteriológico, n 5, 1150-082 Lisboa, Portugal; sara.dias@nms.unl.pt; 3 EpiSaúde Sociedade Científica, 7005-837 Évora, Portugal; 4 Faculdade de Ciências da Nutrição e Alimentação da Universidade do Porto, 4200-465 Porto, Portugal; pedrograca@fcna.up.pt; 5 Programa Nacional para a Promoção da Alimentação Saudável, Direção-Geral da Saúde, 1049-005 Lisboa, Portugal; 6 Escola Superior de Saúde do Instituto Politécnico de Leiria, Unidade de Investigação em Saúde (UI), 2411-901 Leiria, Portugal; 7 NOVA Information Management School, Universidade Nova de Lisboa, 1070-312 Lisboa, Portugal; JMM@novaims.unl.pt (J.M.M.); psc@novaims.unl.pt (P.S.C.); 8 Serviço de Reumatologia do Hospital Egas Moniz—Centro Hospitalar Lisboa Ocidental (CHLO-E.P.E.), 1349-019 Lisboa, Portugal; 9 Instituto de Saúde Pública da Universidade do Porto, 4050-091 Porto, Portugal; carlal@med.up.pt; 10 Department of Preventive Medicine and Public Health, University of Navarra, 31008 Pamplona, Spain; mamartinez@unav.es; 11 Escola Nacional de Saúde Pública, Universidade Nova de Lisboa, 1600-560 Lisboa, Portugal; 12 Unidade de Reumatologia—Centro Hospitalar Universitário Lisboa Central (CHULC-Hospital Curry Cabral), 1169-050 Lisboa, Portugal

**Keywords:** Mediterranean diet, MEDAS, FFQ, telephone, nutrition, epidemiology, Portugal

## Abstract

A 14-Item Mediterranean Diet Adherence Screener (MEDAS) questionnaire was developed and validated in face-to-face interviews, but not via telephone. The aims of this study were to evaluate the validity and reliability of a telephone-administered version of the MEDAS as well as to validate the Portuguese version of the MEDAS questionnaire. A convenience community-based sample of adults (*n* = 224) participated in a three-stage survey. First, trained researchers administered MEDAS via a telephone. Second, the Portuguese version of Food Frequency Questionnaire (FFQ), and MEDAS were administered in a semi-structured face-to-face interview. Finally, MEDAS was again administered via telephone. The telephone-administered MEDAS questionnaire was compared with the face-to-face-version using several metrics. The telephone-administered MEDAS was significantly correlated with the face-to-face-administered MEDAS [r = 0.805, *p* < 0.001; interclass correlation coefficient (ICC) = 0.803, *p* < 0.001] and showed strong agreement (k = 0.60). The MEDAS scores that were obtained in the first and second telephone interviews were significantly correlated (r = 0.661, *p* < 0.001; ICC = 0.639, *p* < 0.001). The overall agreement between the Portuguese version of MEDAS and the FFQ-derived Mediterranean diet adherence score had a Cohen’s k = 0.39. The telephone-administered version of MEDAS is a valid tool for assessing the adherence to the Mediterranean diet and acquiring data for large population-based studies.

## 1. Introduction

Food consumption assessment is one of the main challenges for nutritional epidemiology. The more closed to “gold standard” method for food consumption assessment involves multiple food diaries [1,2]. The utilization of these assessment tools is expensive and time-consuming, in terms of both data collection and analysis. Diet quality indices have been proposed as alternative tools that can be used to assess population dietary habits, and several such indices have been previously validated [3,4,5,6,7,8].

One of the most relevant diet indices is focused on adherence to a Mediterranean diet, as this diet is considered to be one of the healthier dietary patterns. The Mediterranean diet has been associated with several health outcomes, namely showing risk reduction on cardiovascular disease in high-risk individuals [9]. The evidence regarding the health benefits of the Mediterranean diet is consistent [9,10]. Thus, the assessment of adherence to this dietary pattern might be useful for health and medical research. The Mediterranean Diet Adherence Screener (MEDAS) is a questionnaire that is composed of 14 items that probe compliance with this dietary pattern [11,12]. This dietary quality index, which is administered in a face-to-face interview, was validated for the Spanish population [11], and more recently in other countries, such as Germany [13,14]. However, MEDAS questionnaire was not yet validated for the Portuguese population. Portugal as country where Mediterranean diet was nominated World’s Intangible Cultural Heritage by UNESCO and where this dietary pattern should be preserved, need to have validated tools to assess population adherence to Mediterranean diet.

For use in epidemiology studies and public health initiatives, the administration of dietary recall tools via new methods, such as telephone interviews and web-based interviews, might be valid alternatives, as these data collection methods are less expensive and time-consuming than face-to-face interviews. Indeed, studies have confirmed that, for certain dietary recall analyses, telephone interview methods are effective and valid and the results are similar to those obtained with face-to-face interviews [15].

Based on this information, the aims of this study were to evaluate the validity and reliability of a telephone-administered version of the MEDAS, as well as to validate the Portuguese version of the MEDAS questionnaire.

## 2. Materials and Methods

### 2.1. Study Design and Population

**Figure 1:**
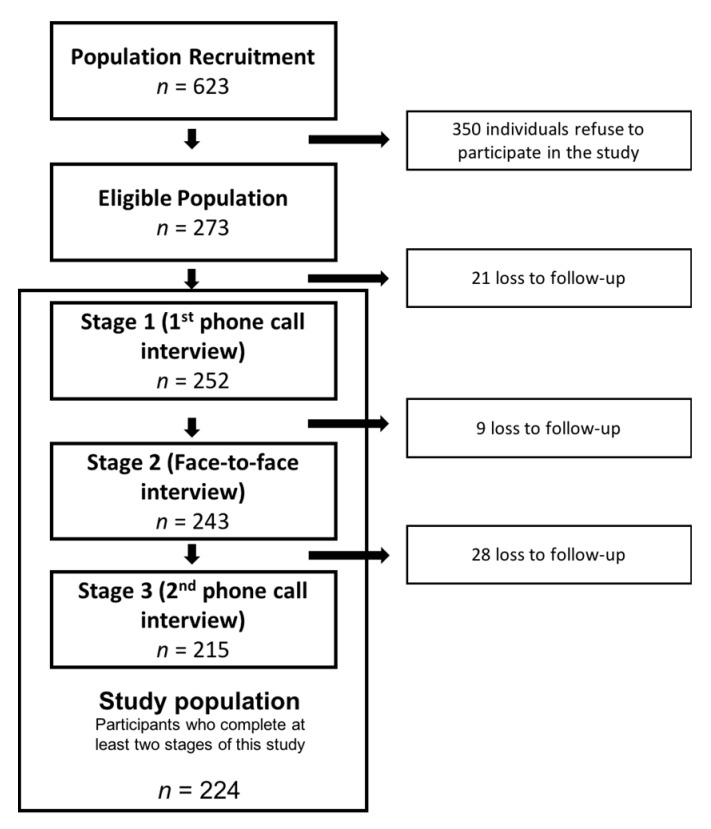
Flowchart of the validation study of a telephone-administered version of the Mediterranean Diet Adherence Screener (MEDAS) questionnaire in the adult Portuguese population.

For this study, we used a convenience population sample from the urban center of Lisbon. The final analysis included 224 individuals that were recruited from two different settings: (a) primary healthcare centers and (b) NOVA Medical School. The sample size was calculated based on the mean difference between the Mediterranean diet adherence score that was obtained by the MEDAS questionnaire and the Mediterranean diet adherence score obtained by the Food Frequency Questionnaire (FFQ; MEDAS score: 8.68 ± 1.90 vs. FFQ score: 8.43 ± 1.73), as described in the validation study for the MEDAS questionnaire for the Spanish population [11]. A sample size of 286 was estimated in order to ensure a statistical power of 80% (β = 0.2) and a significance level of 0.05 (α = 5%). For the recruitment phase, we increased the sample size by 50% to account for the estimated drop-out rate. The sample was also stratified by sex and age group to have a sample that represents the characteristics of the adult Portuguese population. The number of participants in each group was proportional to the real distribution of the population, based on data from the CENSUS 2011. Figure 1 describes the flowchart of this study.

The individuals who agreed to participate provided written informed consent. The inclusion criteria were the explicit agreement to participate in the study and age of at least 18 years. The exclusion criteria were the inability to answer the questionnaire due to the absence of phone contact, language barriers, or cognitive impairment. During the recruitment phase, a structured questionnaire that queried sociodemographic data (age, sex, years of education, income, household composition, professional status, and marital status) was administered. The participants were informed that they would receive a phone call from the research team for an interview. After recruitment, the participants were contacted by phone after approximately seven days.

This study included three stages. Stage 1 involved a phone call interview for the administration of the MEDAS questionnaire. Stage 2 involved a face-to-face interview for MEDAS questionnaire and the FFQ, and Stage 3 involved a phone call interview for repeat administration with the MEDAS questionnaire. The participants who completed the first phone call interview were invited to return to the primary healthcare setting/NOVA Medical School for the administration of the questionnaire via a face-to-face interview. The participants who attended this face-to-face interview were informed that they would be contacted again by phone to for a repeat application of the MEDAS questionnaire to test the reliability of the data (correlation of test and retest). The new contact occurred approximately 15 days after the face-to-face interview. The number of days between the recalls varied per participant; however, to ensure that all of the questionnaires reflect the same reference period, the dietary data collection was carried out during a period of four weeks. Moreover, the length of time between the different stages of data collection was also defined in order to reduce the influence of participants’ memory bias during the different interviews.

For phone calls, the interviewers were trained to administer the MEDAS questionnaire in a standardized way. After a maximum of six phone call attempts on different dates, a telephone appointment was made for the interview. During Stage 2, the FFQ was administered by trained nutritionists. Participants who failed to complete at least two stages of this study were excluded from the analyses.

### 2.2. Dietary Assessment

Adherence to a Mediterranean diet was assessed while using the MEDAS questionnaire from the PREDIMED study [11,12]. The English version of the MEDAS questionnaire was translated into Portuguese while using the “forward-backward” procedure. This questionnaire is comprised of 14 questions related to food intake habits and frequency of consumption of foods that are typical and non-typical of the Mediterranean diet. Responses that were favorable to the adoption of the Mediterranean diet were scored as 1 point, while responses that were unfavorable were scored as 0. The final score ranged between 1 and 14, and a MEDAS score greater than 9 indicates high adherence to the Mediterranean diet [11,12]. We used the data from the MEDAS administered via face-to-face interviews in Stage 2 as the reference for validation of the telephone-administered interview.

The semi-quantitative FFQ was also administered to all participants in this study during Stage 2 via a face-to-face interview. This FFQ was previously developed and validated for the Portuguese population by researchers from the Institute of Public Health, University of Porto [16,17], and it is composed by 82 food items, including nine categories of frequency responses, ranging from “never” to “six or more times per day”. Food quantification and portion-size estimation were based on a food photography manual and on household measures. Nutrient intake data were obtained with the software Food Processor Plus based on values from the US Department of Agriculture and from the composition of typical Portuguese foods that were included in this software. Data regarding food intake obtained by the FFQ were grouped and recodified according to each item of the MEDAS (Table 1). Furthermore, the dietary assessment data from the MEDAS were validated via comparison with the data that were obtained from the FFQ.

### 2.3. Statistical Analysis

Data were assessed via descriptive statistics, including absolute frequencies and percentages for categorical variables and mean and standard deviation (SD) for quantitative variables. We first assessed the agreement between the MEDAS questionnaire in the Portuguese adult population and the FFQ. Kappa (k) statistics were used to determine the agreement between the responses that were obtained for each of the 14 items derived from the MEDAS and from the FFQ. For validation, k values > 0.4 indicated significance [18]. Moreover, the Pearson correlation coefficient and the interclass correlation coefficient (ICC) were calculated in order to analyze the relationship between the scores of the two methods. Additionally, a multivariate linear regression was performed to analyze the association between MEDAS items and the consumption of food groups and nutrients that may be integral characteristics of the Mediterranean diet. Food and nutrient intake recorded on the FFQ was analyzed according to the quintile distribution of MEDAS score administered via face-to-face interview.

We examined the agreement between two repeated measures to evaluate the reproducibility of the MEDAS questionnaire. An analysis of the test-retest was performed using generalized linear models for continuous variables and logistic regression, adjusted for age, sex, and educational level, for categorical variables to measure associations between MEDAS scores obtained by telephone interview in Stage 1 and the MEDAS scores obtained by telephone interview in Stage 3. The same statistical analyses were used to evaluate the association between MEDAS performed by telephone interview in Stage 1 and by MEDAS performed by face-to-face interview for the validation of the telephone-administered method. All of the statistical analyses were performed while using STATA IC version 15 (StataCorp, 2017, Stata Statistical Software: Release 15. College Station, TX: StataCorp LLC).

### 2.4. Ethical Procedures and Personal Protection

This study was reviewed and approved by the National Committee for Data Protection and by NOVA Medical School Ethics Committee (n. 31/2016/CEFCM). All of the participants provided informed consent in accordance with the principles that were established by the Helsinki Declaration revised in 2013 in Fortaleza [19].

## 3. Results

We enrolled 273 Portuguese adults for this study (Figure 1). Table 2 describes the socioeconomic and demographic characteristics of this group. No significantly differences were found in the Mediterranean diet adherence according to socioeconomic characteristics, namely according to educational level, household income, and employment status. The association between the Mediterranean diet adherence score obtained from the FFQ and that obtained from the MEDAS questionnaires were calculated, and absolute agreement of individual component scoring between the MEDAS and the FFQ was determined while using k statistics. A fair agreement (0.21 ≤ k ≤ 0.40) was detected for 64.2% of the questionnaire items, while a moderate agreement was detected for 14.3% of the items. Additionally, good and excellent agreement was detected for 14.3 and 7.1% of the questionnaire items, respectively. Higher agreement was detected for “dink wine” (k = 0.84), “nut consumption” (k = 0.65), and “sugar-sweetened beverages” (k = 0.63). A lower agreement was observed for “butter, margarine, or cream consumption” (k = 0.11), “olive oil as a principal source of fat” (k = 0.25), “quantity of olive oil” (k = 0.23), and “consumption of foods sautéed in olive oil” (k = 0.09) (Table 3). In general, the MEDAS questionnaire score indicated a greater adherence to the Mediterranean diet than did the FFQ (7.29 ± 2.15 vs. 7.16 ± 1.85, *p* < 0.0001, respectively).

The relative and absolute agreements between the MEDAS questionnaire administered via face-to-face interview and the FFQ-derived Mediterranean diet adherence score were significant (r = 0.641, *p* < 0.0001; ICC = 0.634, *p* < 0.0001; k = 0.39 95% CI 0.27–0.51). We also analyzed the food and nutrient intake recorded on the FFQ according to the quintile distribution of MEDAS. All of the associations between MEDAS score and food and nutrient intake recorded on the FFQ were as expected. The consumption of fruits, vegetables, olive oil, fish, and nuts increased with higher MEDAS scores. On the other hand, the consumption of sugar-sweetened beverages and red meat and sausages decreased as the MEDAS score increased. Regarding nutrient intakes, the intake of fiber, vitamin C, vitamin E, folic acid, and omega-3 and -6 fatty acids increased with higher MEDAS scores (Table 4).

The MEDAS scores that were obtained at different time points were mainly concordant. Relative and absolute agreements between MEDAS scores obtained via face-to-face interviews and those obtained by telephone (r = 0.805, *p* < 0.0001; ICC = 0.803, *p* < 0.0001; k = 0.597 95% CI 0.492–0.702) and between MEDAS scores that were obtained by telephone in Stage 1 and those obtained by telephone in Stage 3 (r = 0.661, *p* < 0.0001; ICC = 0.639, *p* < 0.0001; k = 0.572, 95% CI 0.461–0.683) were also significant. General linear models and logistic regression were also used to assess the association between the answers obtained through the face-to-face MEDAS questionnaire and the MEDAS questionnaire administered by telephone and between the MEDAS questionnaire administered by telephone in Stage 1 and that administered in Stage 3. We did not find any significant differences in the majority of the MEDAS questionnaire items between the three measurements (Table 5).

## 4. Discussion

In this study, we demonstrated that the telephone administered MEDAS questionnaire is an accurate and reliable tool for accessing Mediterranean diet adherence in the adult population in Portugal. The scores from the telephone administered MEDAS were significantly correlated with the scores that were from the face-to-face administration of the MEDAS. Furthermore, the MEDAS score obtained via a telephone interview in Stage 1 and that obtained via a telephone interview in Stage 3 were also significantly correlated. The two-telephone administered MEDAS questionnaire results were significantly concordant for the majority of MEDAS items. Lower agreement was found for the question “How many times per week do you consume boiled vegetables, pasta, rice, or other dishes with a sauce of tomato, garlic, onion, or leeks sautéed in olive oil?”. The differences found may be related to the different methodologies that were used to help participants to quantify food portions. In the face-to-face interview, food photographs that are helpful in improving the accuracy of food quantification by surveyed individuals were used. When we examined the results from participants that were categorized as having a strong adherence to the Mediterranean diet; however, no significant differences were found between the MEDAS administered by telephone interview and the MEDAS administered via a face-to-face interview.

Furthermore, we uncovered a weak association between data from MEDAS and data obtained from the FFQ, although, for 35.7% of the MEDAS questions, we found moderate to excellent levels of agreement. We based our conclusions of acceptable correlation on the determination of a Cohen’s k ≥ 0.4, as suggested by Cade et al. [18]. Importantly, the level of agreement found in this study was not different from that obtained in MEDAS validation studies for Spain and Germany [11,13] or in other studies that compared data from diet quality indices and data from FFQ [20,21,22].

In our study, lower agreement values were found for the questions regarding “butter, margarine, or cream consumption” (k = 0.11), “olive oil as a principal source of fat” (k = 0.25), “quantity of olive oil” (k = 0.23), and “consumption of foods sautéed in olive oil” (k = 0.09). These differences are likely due to the method of dietary data collection via the FFQ. For example, the difference in responses to “consumption of foods sautéed in olive oil” is not surprising, as cooking methods are not evaluated by the FFQ. Using the FFQ data, we have estimated the answer to this question by the consumption of tomato, onion, and olive oil. Moreover, the items with lower levels of agreement between the MEDAS and the FFQ in our findings are consistent with the results obtained in MEDAS validation studies for Spain and Germany [11,13]. As with all dietary intake studies, several factors, such as respondent’s memory and their ability to quantify consumption, could potentially underlie these differences.

The MEDAS questionnaire significantly overestimated the mean score for adherence to the Mediterranean diet when compared with the FFQ-derived score, consistent with the results that were obtained in the MEDAS validation study for Spain [11] (7.29 ± 2.15 vs. 7.16 ± 1.85, *p* < 0.0001 respectively). Nevertheless, we found a good absolute agreement between the score obtained by the MEDAS questionnaire and the score obtained from the FFQ (r = 0.641, *p* < 0.0001; ICC = 0.634, *p* < 0.0001).

We tested the association between MEDAS score and the consumption of these foods obtained by FFQ, as the Mediterranean diet is characterized by high consumption of vegetables, fruits, pulses, nuts, fish, and olive oil and by low consumption of red meat and sausages and of sugar-sweetened beverages. Additionally, we also tested this association for nutrient intake. We found that the consumption of foods that are typically encouraged in the Mediterranean diet increases as the Mediterranean diet adherence score obtained from MEDAS increased. Nutrient intake was also associated with the adherence to the Mediterranean diet, as expected. These results are in line with the findings that were obtained by Schroder et al. in a validation of the MEDAS in Spain [11].

Considering the health benefits of the Mediterranean diet and the Portuguese trends for lower adherence to the Mediterranean diet, the promotion of this dietary pattern will be helpful to facilitate strategies for the treatment and prevention of chronic diseases. Specifically, the Portuguese National Program for Healthy Eating Promotion of the Directorate-General of Health established the promotion of the Mediterranean diet as a priority and, in 2016, this organization published a new food guide for the Portuguese population based on the Mediterranean diet principles—Mediterranean Diet Wheel [23]. Moreover, national food and nutrition policies are committed to the development of food and nutrition surveillance and, in Portugal, adherence to this dietary pattern was recommended by the national health authority to serve as an indicator that should be monitored in order to evaluate trends in the dietary consumption of the Portuguese population [24]. Thus, data from this study validate a useful tool, the Portuguese language MEDAS that can be employed to monitor the national food and nutrition policies in Portugal. Moreover, the evidence is consistent with the importance of studying dietary patterns and their association with health outcomes as opposed to analyzing isolated foods or nutrients. Thus, the use of diet quality indices is useful for the collection of data that represent the complexity of the overall diet, taking the synergistic effects of different nutrients and foods into account. In fact, the World Health Organization suggested that, in nutritional epidemiologic studies, population food consumption should be based on eating patterns [25,26,27].

While this study validated a telephone interview-based diet adherence questionnaire for use in the Portuguese population, this study also has some limitations. Although we used an FFQ version that was validated for the Portuguese population, we did not assess dietary intake using the reference method, which could allow a better match and agreement between isolated items. Additionally, one possible reason for the observed poor agreement for some items stems from the difficulty of participants in quantifying food consumption. Indeed, the items that exhibited lower levels of agreement between questionnaire scores corresponded to questions that require more precise food quantification. Moreover, both of the tools used in this study for the assessment of dietary intake were self-reported, which can provide inaccurate data. However, the comparison with the general MEDAS score with food and nutrients form FFQ, showing the expected tendency, supports its validity. An additional limitation could be the small sample size of our study, although it was possible to find significant differences when expected.

The study also had several strengths, including an established methodology, a community-based sample (with age and sex strata distribution according to the Portuguese population), and well-trained nutritionists and researchers that administered the face-to-face and the telephone questionnaires.

The findings of this study will contribute to our understanding of the adherence to the Mediterranean diet worldwide and, ultimately, to promote healthy food behavior. The potential for the administration of the MEDAS via a telephone interview will minimize the time that is needed for dietary data collection, benefitting both researchers and participants. Thus, in the field of nutritional epidemiology, telephone interviews for the collection of dietary intake data may serve as a cost-effective and feasible tool. Additionally, when compared to face-to-face interviews, dietary data collection performed via telephone might offer other advantages. Several studies indicated that subjects are more open and honest in reporting their dietary intake in telephone interviews, reducing the underestimation of consumption or portion size of unhealthy foods, and the overestimation of consumption of healthy foods [15].

In conclusion, data from this study show that the face-to-face and telephone-administered version of the MEDAS questionnaire is a reliable and valid tool to screen diet quality, namely adherence to the Mediterranean diet. This questionnaire is an effective, inexpensive, and easily administered tool that might provide useful data regarding dietary intake in large population-based studies, as well as in clinical trials in Portugal and across the globe.

## Figures and Tables

**Table 1:** MEDAS questions and criteria obtained from Food Frequency Questionnaire (FFQ) data.

MEDAS Question	Criteria to Obtain 1 Point According to FFQ Data
1. Do you use olive oil as the principal source of fat for cooking?	1 point given: if the quantity of olive oil consumed was higher than the quantity of other vegetable oils, margarine, and butter
2. How much olive oil do you consume per day (including that used in frying, salads, meals eaten away from home, etc.)?	1 point given: if the quantity of olive oil consumed was >54 g
3. How many servings of vegetables do you consume per day?	1 point given: if the mean frequency of consumption of all vegetables included in FFQ was ≥2–3 portions per day
4. How many pieces of fruit (including fresh-squeezed juice) do you consume per day?	1 point given: if the mean frequency of consumption of all fruit included in FFQ was ≥2–3 portions per day
5. How many servings of red meat, hamburger, or sausages do you consume per day?	1 point given: if the quantity of beef, veal, pork, lamb, and hamburgers consumed was <100 g
6. How many servings (12 g) of butter, margarine, or cream do you consume per day?	1 point given: if the quantity of butter and margarine consumed was <12 g per day
7. How many carbonated and/or sugar-sweetened beverages do you consume per day?	1 point given: if the mean frequency of sugar-sweetened beverages was <1 portion per day
8. Do you drink wine? How much do you consume per week?	1 point given: if the frequency of wine consumption was <1 portion per day
9. How many servings of pulses do you consume per week?	1 point given: if the frequency of pulses consumption was ≥3–4 portions per week
10. How many servings of fish/seafood do you consume per week?	1 point given: if the mean frequency of consumption of all fish and seafood items of FFQ was ≥2–3 portions per day
11. How many times do you consume commercial (not homemade) pastry such as cookies or cake per week?	1 point given: if the mean frequency of all pastry items of FFQ was ≥2–3 portions per day
12. How many times do you consume nuts per week?	1 point given: if the frequency of consumption of all nuts of FFQ was ≥2–3 portions per week
13. Do you prefer to eat chicken, turkey, or rabbit instead of beef, pork, hamburgers, or sausages?	1 point given: if the quantity of white meat consumed in grams per day (poultry, chicken, rabbit) is higher than the quantity of red meat (beef, veal, pork, lamb, processed meat)
14. How many times per week do you consume boiled vegetables, pasta, rice, or other dishes with a sauce of tomato, garlic, onion, or leeks sautéed in olive oil?	1 point given: if the frequency of consumption of tomato, onion, and olive oil was ≥2 week

**Table 2:** Study participants’ socioeconomic characteristics.

Variables	Participants	
	*n* (%)	95% CI
Age group		
18–29 years	37 (16.5)	12.2–22.0
30–49 years	38 (17.0)	12.6–22.5
30–49 years	36 (16.1)	11.8–21.5
50–59 years	49 (21.9)	16.9–27.8
60–69 years	29 (12.9)	9.1–18.0
70 years or more	35 (15.6)	11.4–21.0
Sex		
Female	129 (57.6)	51.0–63.9
Male	95 (42.4)	36.1–49.0
Educational level		
>12 years	92 (43.2)	36.7–50.0
10–12 years	74 (34.7)	28.6–41.4
5–9 years	16 (7.5)	4.6–11.9
0–4 years	31 (14.6)	10.4–20.0
Marital status		
Single	57 (25.4)	20.1–31.6
Married	109 (48.7)	42.1–55.2
Divorced	24 (10.7)	7.3–15.5
Widow	22 (9.8)	6.5–14.5
Consensual union	12 (5.4)	3.1–9.2
Household income		
<500 €	8 (4.4)	2.2–8.6
500–999 €	43 (23.8)	18.1–30.5
1000–1499 €	49 (27.1)	21.1–34.0
1500–1999 €	22 (12.2)	8.1–17.8
2000–2499 €	15 (8.3)	5.0–13.3
>2500 €	44 (24.3)	18.6–31.1
Household composition		
1 person	31 (13.9)	9.9–19.1
2 people	72 (32.3)	26.4–38.7
3 people	61 (27.4)	21.9–33.6
≥4 people	59 (26.5)	21.1–32.7
Employment status		
Student	26 (11.7)	8.0–16.6
Employed	123 (55.2)	48.5–61.6
Unemployed	20 (9.0)	5.8–13.5
Retired	48 (21.5)	16.6–27.4
Other	6 (2.7)	1.2–5.9

Sample size is not constant due to missing data: Age (*n* = 224); Sex (*n* = 224); Educational level (*n* = 224); Marital status (*n* = 224); Household income (*n* = 181); Household composition (*n* = 223); Employment status (*n* = 223).

**Table 3:** Agreement between MEDAS and FFQ data.

MEDAS Questions ^1^	MEDAS Performed Face-to-Face (%) ^2^	FFQ (%) ^3^	k	95% CI ^4^
1	216 (96.4)	211 (94.2)	0.25	−0.01; 0.51
2	68 (30.4)	15 (6.7)	0.23	0.11; 0.34
3	91 (40.6)	46 (20.5)	0.27	0.15; 0.39
4	74 (33.0)	43 (19.2)	0.33	0.21; 0.46
5	151 (67.4)	195 (87.1)	0.33	0.20; 0.45
6	103 (46.0)	208 (92.9)	0.11	0.05; 0.17
*7*	184 (82.1)	189 (84.4)	0.63	0.50; 0.77
8	45 (20.1)	40 (17.9)	0.84	0.75; 0.93
9	65 (29.0)	64 (28.6)	0.58	0.47; 0.70
10	135 (60.3)	156 (69.6)	0.31	0.18; 0.44
11	76 (33.9)	67 (29.9)	0.40	0.27; 0.52
12	71 (31.7)	80 (35.7)	0.65	0.55; 0.76
13	165 (73.7)	147 (65.6)	0.48	0.35; 0.60
14	188 (83.9)	143 (63.8)	0.09	−0.03; 0.21
Adherence to Mediterranean diet (Score)	108 (48.2)	102 (45.5)	0.39	0.27; 0.51

^1^ The list of MEDAS questions can be found in Table 1. ^2^ Percentage of participants scoring 1 on the MEDAS. ^3^ Percentage of participants scoring 1 on the FFQ. ^4^ 95% CI of k. *n* = 224.

**Table 4:** Food and nutrient intake recorded on the FFQ according to quintile distribution of MEDAS score administered via face-to-face interview.

	1st Quintile	2nd Quintile	3rd Quintile	4th Quintile	5th Quintile	*p*-Linear Trend
**Foods**						
Fruits (g)	251.0 (169.3–332.8)	262.8 (215.9–309.7)	314.6 (253.1–376.2)	321.4 (265.7–377.1)	383.5 (339.1–427.9)	<0.001
Vegetables (g)	128.9 (69.1–188.7)	181.0 (146.7–215.3)	205.0 (160.0–250.1)	288.2 (247.4–328.9)	292.1 (259.6–324.6)	<0.001
Olive oil (g)	14.9 (7.3–22.5)	24.1(19.7–28.4)	21.2 (15.4–26.8)	25.1 (19.9–30.3)	26.6 (22.5–30.7)	0.09
Fish (g)	60.2 (39.8–80.6)	73.8 (62.1–85.5)	88.8 (73.5–104.2)	86.5 (72.6–100.4)	99.9 (88.8–111.0)	<0.001
Nuts (g)	8.7 (−6.8–24.3)	12.4 (3.5–21.3)	21.0 (9.2–32.7)	23.8 (13.2–34.4)	33.3 (24.8–41.8)	0.01
Sugar-sweetened beverages (g)	303.5 (227.2–379.7)	95.4 (51.7–139.1)	84.4 (27.1–141.8)	63.4 (11.5–115.3)	55.1 (13.7–96.5)	<0.001
Red meat and sausages (g)	83.7 (62.4–104.9)	73.5 (61.3–85.7)	49.7 (33.8–65.8)	47.7 (33.2–62.2)	41.0 (29.5–52.5)	<0.001
**Nutrients**						
Cholesterol (mg)	370.8 (309.3–432.2)	390.7 (355.5–426.0)	370.4 (324.1–416.6)	379.9 (338.0–421.7)	349.7 (316.3–383.1)	0.56
Fibre (g)	22.1 (19.1–25.2)	23.9 (22.5–25.6)	27.2 (24.9–29.5)	28.6 (26.6–30.7)	32.4 (30.8–34.1)	<0.001
Vitamin C (mg)	119.5 (89.8–149.1)	129.1 (112.1–146.1)	146.1 (123.7–168.4)	173.6 (153.4–193.8)	193.6 (177.5–209.7)	<0.001
Vitamin E (mg)	9.6 (8.0–11.2)	11.9 (11.0–12.9)	12.4 (11.1–13.6)	14.0 (12.9–15.1)	14.9 (14.0–15.7)	<0.001
Folic acid (µg)	319.2 (272.2–366.2)	328.5 (301.6–355.5)	378.3 (342.9–413.7)	377.5 (345.5–409.6)	435.6 (410.1–461.2)	<0.001
Omega-3 fatty acid (g)	1.4 (1.2–1.6)	1.7 (1.6–1.8)	1.6 (1.4–1.7)	1.7 (1.5–1.8)	1.7 (1.6–1.8)	0.03
Omega-6 fatty acid (g)	11.3 (9.4–13.2)	12.9 (11.8–14.0)	12.4 (11.0–13.8)	13.5 (12.2–14.8)	13.8 (12.8–14.8)	0.17

Values are expressed as means and 95% CI. MEDAS score administered via face-to-face interview was divided into quintiles: 1st quintile, *n* = 20; 2nd quintile, *n* = 61; 3rd quintile, *n* = 37; 4th quintile, *n* = 42; 5th quintile, *n* = 64. *p*-linear trend adjusted for sex, age, educational level and energy intake.

**Table 5:** Agreement between MEDAS performed in the three different stages (MEDAS administered by telephone vs. MEDAS administered via face-to-face interview and MEDAS administered by telephone in Stage 1 vs. MEDAS administered by telephone in Stage 3).

MEDAS Questions ^1^	MEDAS Administered by Telephone vs. MEDAS Administered via Face-to-Face Interview (*n* = 224)			MEDAS Administered by Telephone in Stage 1 vs. MEDAS Administered by Telephone in Stage 3 (*n* = 215)		
	Mean ± SD Telephone	Mean ± SD Face-to-Face	*p*-Value	Mean ± SD Telephone 1	Mean ± SD Telephone 2	*p*-Value
**1**	216 (96.4%)	216 (96.4%)	1.00	216 (96.4%)	207 (97.6%)	0.43
**2**	2.69 ± 1.56	2.84 ± 1.74	0.04	2.69 ± 1.56	2.72 ± 1.50	0.22
**3**	1.52 ± 0.74	1.59 ± 0.79	0.08	1.52 ± 0.74	1.51 ± 0.66	0.95
**4**	2.05 ± 1.19	2.15 ± 1.23	0.02 *	2.05 ± 1.19	2.06 ± 1.22	0.73
**5**	0.41 ± 0.55	0.43 ± 0.55	0.48	0.41 ± 0.55	0.38 ± 0.51	0.28
**6**	0.64 ± 0.67	0.68 ± 0.67	0.01 *	0.64 ± 0.67	0.59 ± 0.61	0.88
***7***	0.26 ± 0.69	0.27 ± 0.66	0.09	0.26 ± 0.69	0.28 ± 0.80	0.83
**8**	2.76 ± 4.71	2.84 ± 4.56	0.76	2.76 ± 4.71	2.79 ± 4.83	0.03 *
**9**	1.83 ± 1.38	2.01 ± 1.49	0.09	1.83 ± 1.38	1.71 ± 1.41	0.02 *
**10**	3.35 ± 1.78	3.29 ± 1.93	0.05	3.35 ± 1.78	3.39 ± 1.76	0.49
**11**	3.09 ± 2.9	2.91 ± 2.44	0.09	3.09 ± 2.9	2.84 ± 2.60	0.53
**12**	1.75 ± 2.25	1.85 ± 2.16	0.51	1.75 ± 2.25	1.83 ± 2.18	0.96
**13**	159 (71.0%)	165 (73.7%)	0.85	159 (71.0%)	154 (72.6%)	0.82
**14**	3.37 ± 2.50	3.92 ± 2.64	<0.001 *	3.37 ± 2.50	3.56 ± 2.74	0.30
**Score**	7.15 ± 2.03	7.29 ± 2.15	0.12	7.15 ± 2.03	6.80 ± 2.52	0.01 *
**Adherence to Mediterranean diet**	44.2 (%)	48.2 (%)	0.17	44.2 (%)	45.3 (%)	0.93

^1^ The list of MEDAS questions can be found in Table 1. For questions 2, 3, 4, 5, 6, 7, 8, 9, 10, 11, 12, 13, 14 and score, *p*-value is based on generalized linear models, adjusted for age, sex and educational level. For questions 1, 13, and for adherence to Mediterranean diet, *p*-value is based on logistic regression adjusted for age, sex and educational level. SD—standard deviation. * *p*-value ≤ 0.05.
